# Sarcomatoid carcinoma of the renal pelvis: A case report

**DOI:** 10.3892/ol.2014.2240

**Published:** 2014-06-11

**Authors:** XIQUAN TIAN, JIYU ZHAO, YUE WANG, NIANZENG XING

**Affiliations:** 1 Department of Urology, Beijing Chao-Yang Hospital, Capital Medical University, Beijing 100020, P.R. China; 2 Department of Pathology, Beijing Chao-Yang Hospital, Capital Medical University, Beijing 100020, P.R. China

**Keywords:** sarcomatiod carcinoma, renal pelvis, urothelial tumor

## Abstract

Sarcomatoid carcinoma is a high-grade malignant neoplasm which exhibits morphological and/or immunohistochemical evidence of bidirectional epithelial and mesenchymal differentiation. Sarcomatoid carcinoma occurring in the upper urinary tract is rare. The present study reports a case of primary sarcomatoid carcinoma of the renal pelvis. A 49-year-old female patient was admitted to Beijing Chao-Yang Hospital for experiencing two weeks of intermittent hematuria. A computed tomography scan revealed a mass of 2 cm in diameter in the left renal pelvis. A retroperitoneoscopic nephroureterectomy combined with a bladder cuff excision was performed, and the final pathological diagnosis was sarcomatoid carcinoma of the renal pelvis. The patient did not receive systemic chemotherapy and radiotherapy. Regular follow-up was performed for 30 months, and there was no evidence of tumor local recurrence or distant metastasis.

## Introduction

Sarcomatoid carcinomas are malignant neoplasms that demonstrate epithelial and mensenchymal differentiation. The tumor can arise from almost any organ with an epithelial component, including the breast, larynx, oral cavity, esophagus, female genital tract and bladder. Sarcomatoid urothelial carcinomas less commonly arise in urinary tract, and account for ~0.3% of all urothelial carcinomas (1). Sarcomatoid carcinoma is a high-grade, biologically aggressive tumor with a poor prognosis. At the time of diagnosis, nodal and distant metastases are common and the majority of patients succumb to the disease within two years (2). Currently, surgical resection remains the first-line treatment. Sarcomatoid carcinoma of the renal pelvis is extremely rare (3–9); only ten well-illustrated cases have been reported since the first true case reported in 1984 (3). The clinical-pathologic characteristics and potential therapeutic strategies of this tumor are poorly understood due to the lack of large sample data. In April 2011, the Urology Department of Beijing Chao-Yang Hospital affiliated with Capital Medical University (Beijing, China) admitted a patient with primary sarcomatoid carcinoma of the renal pelvis. In the present study, the clinicopathological details and the prognosis of this case are discussed according to the associated literature. Patient provided written informed consent.

## Case report

**Figure 1:**
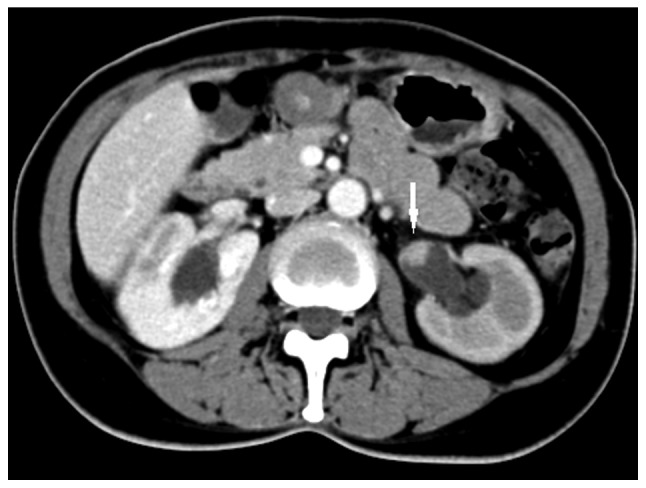
Abdominal computed tomography scan demonstrates a mass in the left renal pelvis.

A 49-year-old female patient presented to the Department of Urology, Beijing Chao-Yang Hospital having experienced intermittent gross hematuria for the past two weeks. Physical examination indicated percussion pain in the left kidney region. An abdominal computed tomography (CT) scan was performed and revealed a 2 cm in diameter space-occupying lesion in the left renal pelvis (Fig. 1). Cystoscopic examination showed intermittent bloody fluid ejection from the left ureter. Urine cytology did not detect malignant or atypical cells. No distant metastases were detected after the patient underwent a whole-body CT scan. The patient’s past medical history disclosed that a laser endoureterotomy procedure had been applied for treatment of the right ureteral stricture 17 months previously. The preoperative diagnosis of the patient was left renal pelvic carcinoma, and a retroperitoneoscopic nephroureterectomy combined with a bladder cuff excision was performed.

**Figure 2:**
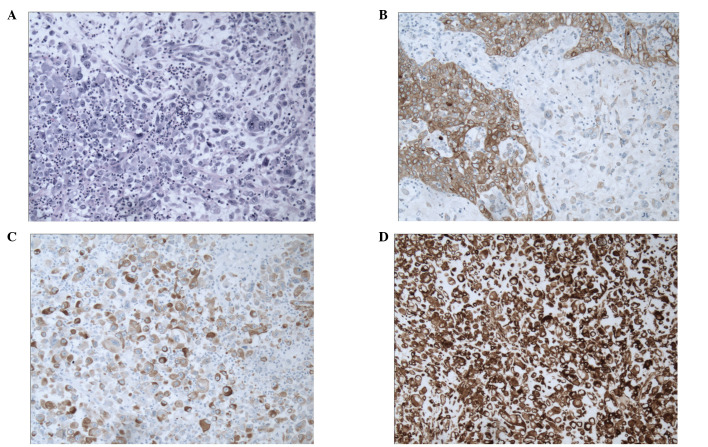
Photomicrographs of the tumor specimen. (A) The sarcomatous area is mainly composed of pleomorphic spindle, round and giant cells (stain, hematoxylin and eosin; magnification, ×200). (B) The transitional cell carcinoma component and the sarcomatoid tumor cells are immunoreactive for cytokeratin (immunostaining; magnification, ×100). (C) Sarcomatoid cells demonstrate positive expression of cytokeratin (immunostaining; magnification, ×200). (D) Vimentin is strongly expressed in the sarcomatoid area (immunostaining; magnification, ×200).

On gross examination, the tumor in the left renal pelvis was ~2 cm in diameter, the section was gray-white in color and there was no obvious bleeding or necrosis. Microscopically, the tumor was composed of high-grade malignant urothelial cells and sarcomatoid differentiated tumor cells. Numerous pleomorphic, giant and multinucleated cells with one or more prominent nucleoli were observed in the sarcomatoid component, and the nuclear chromatin had a coarse granular shape (Fig. 2A). The tumor was confined to the submucosal area; neither renal parenchymal invasion nor local lymph node metastasis was observed. Immunohistochemical studies indicated that the sarcomatoid components were positive for cytokeratin (Fig. 2B and C) and vimentin (Fig. 2D), and were negative for desmin and smooth muscle actin. The final pathological diagnosis was sarcomatoid carcinoma of the renal pelvis (T1N0M0) according to the Union for International Cancer Control 2009 edition of the Tumor Node Metastasis Classification (10). The patient did not receive systemic chemotherapy and radiotherapy due to the organ-confined nature of the tumor. The postoperative recovery of the patient was good. Regular clinical follow-up was conducted for 30 months, and the patient had no evidence of local recurrence or distant metastasis.

## Discussion

The majority of renal pelvic tumors are urothelial tumors; primary sarcomatoid carcinoma of the renal pelvis is clinically rare. From the current data, the age of onset for this disease is usually >50 years and the male-to-female ratio is 2–3:1. Presenting symptoms include gross hematuria, flank pain, an abdominal mass and hydronephrosis, and are often similar to those of urothelial tumors. However, sarcomatoid carcinoma is a higher-grade aggressive epithelial neoplasm compared with urothelial tumors, which are associated with higher tumor stage, frequent metastases at presentation and a poor prognosis (2–9). Commonly, it is almost impossible to establish a correct histopathological diagnosis preoperatively, due to the lack of distinctive features. The diagnosis is often made on the basis of the histological pattern and the immunohistochemical findings, postoperatively.

The differential diagnosis for this tumor includes a variety of diseases, such as carcinosarcoma, primary and secondary spindle cell sarcomas and sarcomatoid renal cell carcinoma. Sarcomatoid carcinoma is easily confused with carcinosarcomas, due to the two tumor types having similar histological morphologies. Immunohistochemical studies are mandatory for the correct diagnosis. Cytokeratins and other epithelial cell markers are specific for cells of epithelial origin, and true sarcomatous components of carcinosarcomas do not stain for epithelial markers. Immunohistochemical staining may also be of help to distinguish sarcomatoid carcinomas from mesenchymal malignancy (6–8). It may be difficult to recognize a sarcomatoid carcinoma as either a primary renal or urothelial tumor. Identification of improved differential characteristics of urothelial cell, as well as demonstration of transitions with *in situ* urothelial carcinoma, may aid the diagnosis (11). When the spindle cell component predominates and there is limited or no morphologic evidence of epithelial differentiation, the immunohistochemical staining of PAX8 and the transcription factor GATA3 may be of particular value (12).

Due to the limited cases reported in the literature, currently, there is no recommended treatment regimen for sarcomatoid carcinoma of the upper urinary tract. When the patient’s condition permits, radical resection is preferred. However, whether radical surgery can prolong patient survival awaits further study (13). Other auxiliary treatments, including systemic chemotherapy and radiotherapy, are only observed in certain case reports, with limited clinical experience (9). More recently, a study showed that the expression of epidermal growth factor receptor (EGFR) was positive in the majority of sarcomatoid carcinomas of the upper urinary tract, suggesting that anti-EGFR molecular targeted therapy may be a promising therapeutic direction in the future (5).

Sarcomatoid carcinoma of the renal pelvis is a highly malignant tumor. A poor prognosis has been reported for the majority of patients and only a few patients have survived for more than two years following diagnosis (4–8). The pathological stage may be an important factor for determining prognosis. Currently, the longest reported survival of an affected patient is >108 months (14). The patient in the present study had a tumor that was confined to the renal pelvis and has had no signs of metastatic spread; therefore, we predict that the present patient may have a superior prognosis. Following radical surgery, at the time of writing, the patient has survived for 30 months without receiving radiotherapy and chemotherapy. Radiological examination has not yet detected either local recurrence or distant metastasis, and regular follow-ups are ongoing.

In conclusion, the clinicopathological features, treatment and prognosis of sarcomatoid carcinoma of the upper urinary tract remains poorly understood and additional data are required to understand this disease.
